# First Two Cases of Conservative Treatment for Extreme Proximal Penile Fracture of the Corpora Cavernosa

**DOI:** 10.1155/2023/5706109

**Published:** 2023-06-10

**Authors:** Alicia Blondeau, Aurélie Grandmougin, Clément Larose, Charles Mazeaud

**Affiliations:** ^1^Department of Urology, Nancy University Hospital, France; ^2^Department of Radiology, Nancy University Hospital, France

## Abstract

Penile fracture is a urological emergency, and surgery is usually recommended to prevent complications. However, proximal locations are scarce and not well investigated. We present two rare penile fractures involving the proximal corpora cavernosa with an original conservative strategy to manage this clinical presentation. Twenty-five- and thirty-eight-year-old men with no previous medical history were admitted to the emergency room for penile trauma during sexual intercourse a few months apart. Both presented with “butterfly pattern” ecchymosis with a palpable hematoma on the perineum. They had no hematuria or voiding dysfunction. Ultrasound found a hematoma and a tear of the proximal corpus cavernosum for the younger one. Then, an MRI confirmed a longitudinal fracture of the right corpus cavernosum for the first patient and left for the second, without urethral injury. In agreement with the patients facing this atypical presentation, we proposed a conservative treatment with analgesics, monitoring, and advice to stop sexual activity for three weeks. After six weeks and four weeks, respectively, we performed a clinical evaluation and a second MRI that found no residual tear or hematoma. The IIEF-5 questionnaire was 24/25 and 25/25. The patients were clinically symptom-free at 8 and 11 months of follow-up. Extreme proximal fracture of the corpus cavernosum can be managed conservatively in selected situations. MRI is useful for decision-making by confirming the diagnosis and location to avoid surgery.

## 1. Introduction

Penile fracture is a urologic emergency first described by Malis in 1924. Most patients are young, and the diagnosis is clinical. Surgery repair is usually recommended to prevent complications. However, proximal locations of fractures on the corpus cavernosum are rare and do not have the same anatomical and clinical characteristics [1]. Therefore, the conservative management of this small subset of injury should be addressed. We describe the two first proximal penile fractures conservatively managed.

## 2. Cases Presentation

Twenty-five- and thirty-eight-year-old men were admitted three months apart to our urologic center. They had the exact mechanism of traumatism: a penile trauma during sexual intercourse with a sharp pain at the root of the scrotum.

**Figure 1:**
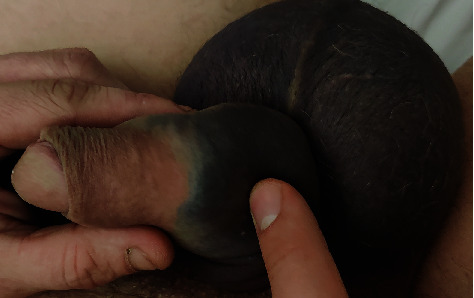
Large scrotal hematoma spreading to the penis.

The youngest had no immediate detumescence of the penile, and a large scrotal hematoma appeared extending to the penis. The patient presented at the emergency department with a “butterfly pattern” of ecchymosis over the perineum and palpable hematoma focused on the fracture (Figure 1). Penoscrotal ultrasound imaging revealed a 6-mm right proximal corpus cavernosum longitudinal tear. He had no hematuria, and his erection was preserved.

**Figure 2:**
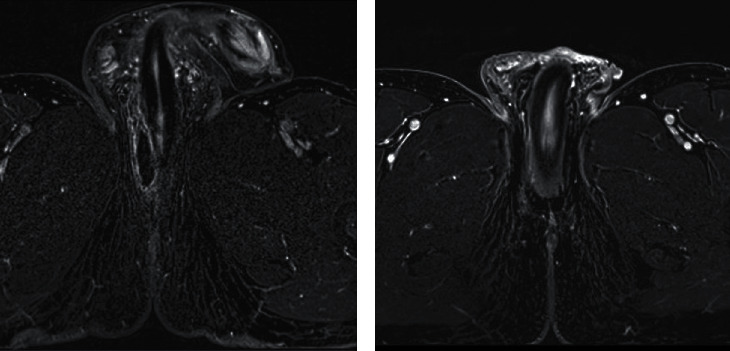
T1-weighted venous time axial pelvis MRI; (a) 8 mm fracture of the corpus cavernosum; (b) complete resolution at six weeks.

**Figure 3:**
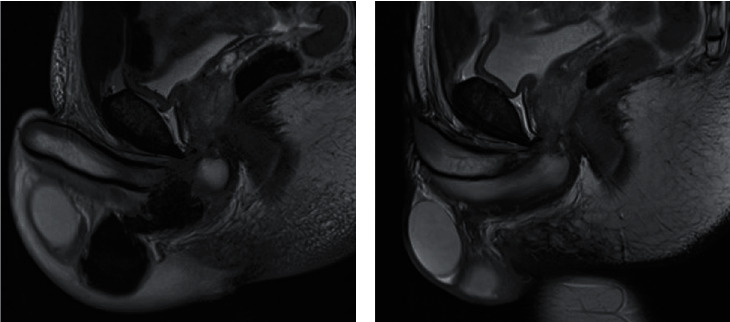
T2-weighted sagittal pelvis MRI; (a) large hematoma spreading in the scrotum; (b) complete resolution at six weeks.

With this atypical presentation of a probable proximal corpus cavernosum fracture, an MRI of the penile was performed the next day and revealed a focal tear of the posterolateral tunica albuginea of the right proximal corpus cavernosum (Figure 2). The tear measured 8 mm with a large hematoma spreading in the scrotum (Figure 3), without urethral injury. In agreement with the patient, we proposed a conservative treatment with analgesics, monitoring, and advice to stop sexual activity for three weeks.

At the 6-week follow-up, the hematoma resorption was nearly complete. The patient could have a rigid erection without difficulty or any penile curvature, pain, or infection. The bruise was no longer visible and palpation revealed no residual swelling. The IIEF-5 questionnaire was out of 24/25. A second MRI was performed to monitor the lesion and revealed complete disappearance of the fracture line, hematoma, or contusion of the corpora cavernosa (Figures 2 and 3).

**Figure 4:**
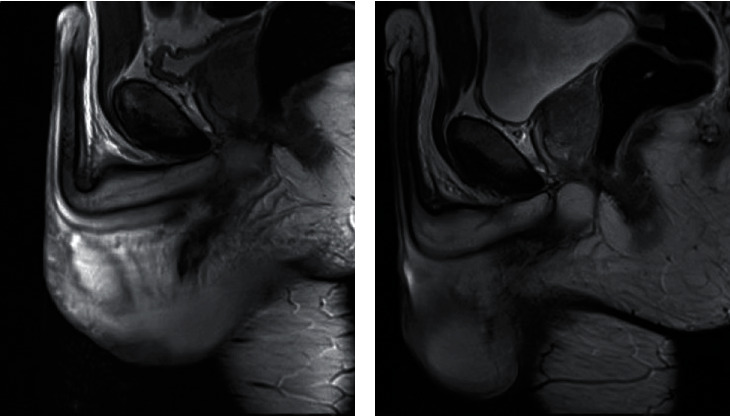
T2-weighted sagittal pelvis MRI; (a) hematoma at the base of the scrotum, (b) complete resolution at one month.

**Figure 5:**
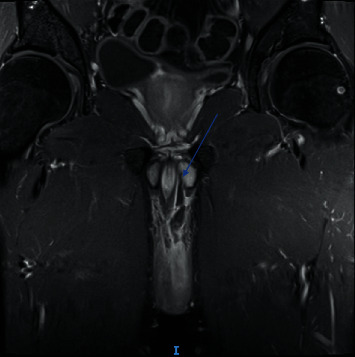
T1-weighted venous time fontal pelvis MRI, focal tear of the medial aspect of the left corpora, adjacent to the penile bulb.

The thirty-eight-year-old patient presented a penile detumescence after a penile-perineal impact in a “doggy style” position. First appeared a scrotal swelling and ecchymosis, and then the “butterfly pattern” emerged. He had no gross hematuria and voiding issue. In the emergency room, an ultrasound was performed, and a hematoma was found, followed by an MRI because of the high probability of a proximal fracture. It revealed a focal longitudinal tear of the medial aspect of the left corpora adjacent to the penile bulb, a hematoma between the bulb and the left corpus cavernosum (Figures 4 and 5), with no urethral lesion. In agreement with the patient, we achieved the same conservative treatment as the first. Twelve hours after the trauma, he already had a rigid erection. Despite a three-week sexual abstinence recommendation, he presented a swelling after sexual intercourse two weeks after the trauma, which regressed during the following days.

At the 1-month follow-up, he related no complications. He was able to have a rigid erection without curvature, pain, or infection. Clinical examination revealed only mild bruising and slight residual induration at the fracture site. The IIEF-5 questionnaire was measured at 25/25. We performed a second MRI and found no tear with total regression of the hematoma (Figure 4).

The patients were called back, respectively, at 8 and 11 months after the trauma; they reported no clinical symptoms, including no erectile dysfunction or any curvature.

## 3. Discussion

We describe the two first patients treated conservatively, with simple outcomes at more than six months of follow-up.

International urological guidelines recommend early surgical repair of penile fractures to reduce the risk of erectile dysfunction and secondary curvature [2–4]. However, none specifically mentions the location of the fracture or atypical clinical presentations. Furthermore, as this condition is rare, the subgroup with proximal fractures is even rarer, as evidenced by the lack of descriptive literature. Thus, the management of extreme proximal fractures remains unclear.

Our conservative attitude was motivated by several reasons. First, our patients did not have an immediate detumescence, and the swelling was perineal without apparent signs of ureteral injury (hematuria and urinary retention). Second, the MRI confirmed that the fracture was not on the mobile penis but on its extremely proximal root. This clinic-radiological description differs from the EAU and AUA recommendations.

The day after the injury, both had a natural and painless semierection after the trauma. The patient and the surgical staff discussed our conservative attitude and the associated risks. The additional arguments to avoid an intervention were the postoperative complications related to the degloving, particularly the penoscrotal or perineal incision. Many surgical complications are described in the literature, such as plaques, nodules, curvature, erectile dysfunction, pain, and infection.

However, a meta-analysis published in 2016 and a recent one in 2020 demonstrated that surgical treatment has fewer complications than conservative management [5, 6]. Unless no analysis was performed to establish a relation between localization of the tear and complications with the conservative treatment, only 5.5% of participants had conservative management, and only four patients had corporal root injuries.

With a more than six-month follow-up, our patients presented no curvature or erectile dysfunction. Four cases of extreme proximal fracture of the corpora cavernosa have been described in the literature to date [1, 7–9], managed surgically. One had a penoscrotal incision, and three had a perineal incision. One patient had penile curvature at the fracture repair side, 3 had no erectile dysfunction at one month [7], two months [9], and six months [1], and no outcome description was available for the last one [8].

Facing these atypical symptoms and the penile probability of fracture, a penile MRI in semiemergency was helpful. It allowed us to confirm and locate the tear. According to Saglam et al., MRI is sensitive to detecting corpora cavernosa fractures of 100%, a specificity of 87.5%, a positive predictive value of 96.7%, and a 100% negative predictive value [10].

MRI is not operator dependent or impacted by the hematoma or pain compared to ultrasound. Moreover, improving the ultrasound experience with such a rare disease is difficult. MRI can, therefore, be used to assess the localization and severity of the rupture and avoid unnecessary degloving of the penis and its complications. The second MRI ensured the fracture disappeared before resuming regular sexual activity in these two exceptional cases. Despite these excellent results, a significant limitation is that MRI is an expensive examination and not always readily available. Indeed, not all trauma centers are provided with an MRI equipment or are not intended to be available in an emergency.

Our patient and the other four in the literature had different sexual positions during the trauma: “on top,” “doggy style,” and two unknown positions. In the literature, the “doggy style” position followed by the “man on top” position seems to be the most frequent cause of penile fracture [11, 12]. However, according to Pruthi et al., the mechanism of extreme proximal corpora cavernosa fracture is different: “longitudinal tears most likely from compression or shearing forces on the corpora at a relatively fixed point, that is near its attachment to the pubis” [1]. This scarce situation makes it difficult to prove a relation between sexual position and extreme proximal corpora cavernosa fracture. However, the hypothesis of a different lesion mechanism supports the idea of a different evolution to healing.

Further studies are needed to assess whether fracture location and longitudinal orientation are determinants of conservative treatment.

We described the first two cases of extreme fracture of the proximal corpora cavernosa treated conservatively without any complications. MRI is useful for decision-making by confirming the diagnosis and location.

## Data Availability

The corresponding author is at your disposal for any further data information.
